# Filter presence and tipping paper color influence consumer perceptions of cigarettes

**DOI:** 10.1186/s12889-015-2643-z

**Published:** 2015-12-22

**Authors:** Richard J. O’Connor, Maansi Bansal-Travers, K. Michael Cummings, David Hammond, James F. Thrasher, Cindy Tworek

**Affiliations:** Department of Health Behavior, Roswell Park Cancer Institute, Buffalo, NY 14263 USA; Department of Psychiatry and Behavioral Sciences, Medical University of South Carolina, Charleston, SC USA; School of Public Health and Health Systems, University of Waterloo, Waterloo, ON Canada; Department of Health Promotion, Education & Behavior, Arnold School of Public Health, University of South Carolina, Columbia, SC USA; Department of Pharmaceutical Systems and Policy, West Virginia University, Morgantown, WV USA

**Keywords:** Tobacco, Perception, Design

## Abstract

**Background:**

Cigarettes are marketed in a wide array of packaging and product configurations, and these may impact consumers’ perceptions of product health effects and attractiveness. Filtered cigarettes are typically perceived as less hazardous and white tipping paper (as opposed to cork) often conveys ‘lightness’.

**Methods:**

This study examined cigarette-related perceptions among 1220 young adult (age 18-35) current, ever, and never smokers recruited from three eastern U.S. cities (Buffalo NY, Columbia SC, Morgantown WV). Participants rated three cigarette sticks: two filtered cigarettes 85 mm in length, differing only in tipping paper color (cork versus white), and an unfiltered 70 mm cigarette.

**Results:**

Overall, the cork-tipped cigarette was most commonly selected on taste and attractiveness, the white-tipped on least dangerous, and the unfiltered on most dangerous. Current smokers were more likely to select white-tipped (OR = 1.98) and cork-tipped (OR = 3.42) cigarettes, while ever smokers more commonly selected the cork-tipped (OR = 1.96), as most willing to try over the other products. Those willing to try the filtered white-tipped cigarette were more likely to have rated that cigarette as best tasting (OR = 11.10), attracting attention (OR = 17.91), and lowest health risk (OR = 1.94). Similarly, those willing to try cork tipped or unfiltered cigarettes rated those as best testing, attracting attention, and lowest health risk, respectively.

**Conclusions:**

Findings from this study demonstrate that consumer product perceptions can be influenced by elements of cigarette design, such as the presence and color of the filter tip.

## Background

Cigarettes are marketed in a wide array of packaging and product configurations designed to appeal to different consumer groups [1]. Aside from packaging, some points of difference between cigarette sticks that are readily apparent to the consumer include the presence or absence of a filter, overall length, diameter, and the color and appearance of tipping paper (which attaches the filter to the tobacco column). Borland and Savvas [2], and also internal marketing research documents [3–6], show that these characteristics of the cigarette itself have promotional effects inasmuch as they drive consumer perceptions about relative product attractiveness, harm, quality, value, and intention to use.

Certain aspects of cigarette design, such as the presence or absence of a filter, influence consumer perception of health risk. Surveys of current and former smokers find that a majority believe cigarettes with filters are safer than those without them [7, 8]. This is consistent with internal tobacco industry research, which showed that the presence of a filter reduced health fears associated with smoking [9]. The cigarette filter rose to prominence in the early 1950s, implying health protection for smokers at a time when the first scientific evidence on the health effects of smoking appeared in the scientific literature and popular press [10, 11]. However, filters do not appear to have a beneficial effect on smoking-related mortality [12–14], and may have played a role in the shift of lung cancer incidence from peripheral squamous cell carcinoma to central adenocarcinoma [14–17]. Filters may also carry health burden in their own right through release of inhalable plastic fibers [10], elasticity of delivery [18, 19], and perhaps making tobacco smoke more mutagenic [20–22]. Filters have also been cited as a source of toxic environmental pollution [23, 24]. There have been calls in some quarters to focus more regulation on filters, including banning the use of vents [19, 25], and extending producer responsibility to pay for their environmental impact [23, 26, 27]. Similarly, cigarettes historically marketed as “light,” which largely relied on ventilated filters to reduce yields [19] have been perceived as less hazardous to health [28–30], and terms such as “light” and “mild have been banned as misleading in a number of countries.

The color of tipping paper can affect sensory perceptions around products, with white tipping paper (commonly used on “light” cigarettes) conveying ‘lightness’ [3–6]. Borland and Savvas [2] note a number of internal tobacco industry studies of “sensation transfer” that reveal how smokers’ perceptions of cigarette attributes are impacted by product appearance, such as length and colors. Their study among Australian smokers showed that cork-tipped products were judged most attractive, highest quality, and strongest in taste relative to others. This effect is consistent with literature on the effects of packaging color on product perceptions of attractiveness, taste, and health risks [31–33].

The current study examined how consumers (both smokers and nonsmokers) perceive differences in health risk, taste, and attractiveness among three common cigarette stick configurations found in manufactured cigarettes sold in the United States – unfiltered, filtered cork-tipped (commonly used for ‘full-flavor’ variants), and filtered white-tipped (commonly used for lower ISO tar variants).

## Methods

Study data come from a field experiment conducted at three sites [Buffalo, NY (*N* = 408); Columbia, SC (*N* = 406); Morgantown, WV (*N* = 406)] in the United States from April-July 2011. The overall study examined the influence of tobacco package shape and size and product configuration on consumer perceptions of appeal and health risks for cigarettes and smokeless tobacco products (Bansal-Travers et al., unpublished). This study was approved by the Institutional Review Boards at the Roswell Park Cancer Institute, the University of South Carolina, and the West Virginia University, to safeguard the rights of all participants. Young adults (*N* = 1220; aged 18-35 years) were recruited from public spaces (e.g., libraries, parks, sporting events, shopping malls) to complete the 20-min experiment and received a $10 gift card for their time and effort. Participants in the current analysis were current (*N* = 589), ever (*N* = 352) and never (*N* = 279) smokers, with current use defined as any use of cigarettes in the previous 30 days. There was no significant difference in the relative proportions of current (22-23 %), ever (27-31 %), or never (47-50 %) smokers across sites (*p* = 0.757). The participants were 67 % non-Hispanic white, 19 % non-Hispanic black, 6 % Hispanic, 55 % male, and 80 % with >12 years education; of the current smokers, 44 % smoked cigarettes daily. Of current smokers, 47 % reported using a brand carrying a Light/Mild descriptor or traditionally associated color (e.g., gold, silver). Current smokers were more likely to be male and white, and less likely to have > 12 years of education (all p values ≤ 0.001).

**Fig. 1:**
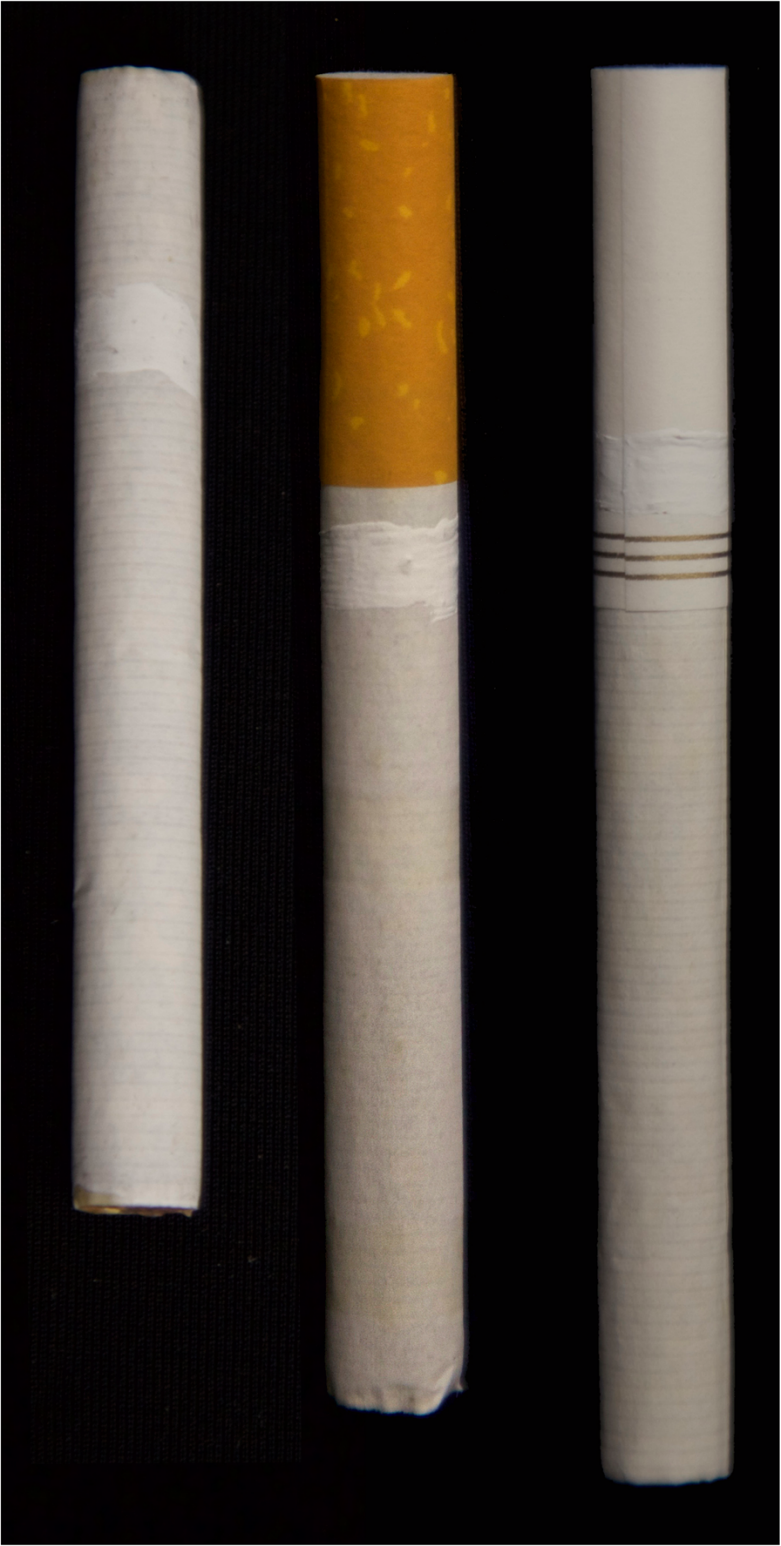
Cigarettes used in demonstration. Left to right: unfiltered, cork-tip, white-tip

Participants were shown three cigarette sticks, with brand name masked (as illustrated in Fig. 1). The two filtered cigarettes were each king sized and of equivalent diameter, differing in tipping paper color (cork versus white). The unfiltered cigarette was 70 mm long and of equivalent diameter to the filtered cigarettes. The six questions participants were asked for the filter tip array included: 1) Which cigarette would you expect to have the best taste?; 2) Which cigarette do you think is most likely to attract your attention?; 3) Which cigarette do you think is most dangerous to your health?; 4) Which cigarette do you think is least dangerous to your health?; 5) Which cigarette would you or someone like you be most willing to try?; and 6) Which cigarette would you or someone like you buy if you were trying to reduce health risks? Response options included selection of one of the three types of cigarettes, ‘no difference’, or ‘don’t know’. Perception of smoking risk was assessed with the question “On a scale of 1 (not at all) to 10 (extremely), how dangerous do you think smoking cigarettes is to your health?” Because responses to this item were severely skewed, we dichotomized at the median (M = 9; therefore categories represent 10 vs 1-9).

### Analysis

Descriptive statistics were used to summarize responses provided for current tobacco use status. One-sample chi squares were used to examine randomness of product selection, and Cramer’s V (which ranges from 0 – 1) was used to examine consistency of selections across questions. Logistic regression models were estimated to determine covariates of willingness to try one cigarette type over the other two types or the ’no difference’ category. Thus, ORs represent the relative odds of selecting a given product as ‘most willing to try’ over the other options. Primary independent variables included cigarette smoking status (current smoker; ever smoker; never smoker) and a set of dummy variables to indicate selection of a particular cigarette type (i.e., white tip, cork tip, unfiltered, with ’no difference’ as the reference group) for questions on product appeal (i.e., tastes best; attracts attention; least dangerous). Regression covariates included study site, age, gender, race/ethnicity (Non-Hispanic White, Non-Hispanic Black, Hispanic, Other), education (coded as up to 12 years versus more than 12 years), and perceived smoking risk.

## Results

Table 1 shows the proportion that selected each cigarette type (unfiltered, filtered cork-tipped, and filtered white-tipped) as most dangerous, least dangerous, and willing to try. Chi-square tests showed that selection patterns of products differed from random (i.e., 25 % per cell; p-values <0.001). Product nominations were correlated, in particular most and least dangerous (V = .567, *p* < 0.001). Because of this strong collinearity, only least dangerous was used in the multivariate model. Taste showed significant relationships to attention (V = .373, *p* < 0.001), and least danger (V = .243, *p* < 0.001). Attention showed a significant relationship to least danger (V = .160, p < 0.001). Table 1 also shows the nominations by smoking status. In most cases, current smokers displayed a different pattern from other participants, in particular less likely to select ‘no difference’ as a response.

**Table 1:** Proportion nominating each product on appeal metrics, overall and by tobacco use status. Chi-square tests indicate deviation of observed selections from the random case (i.e., 25 % per cell)

		White Tip	Cork Tip	Unfiltered	No Difference	
Which cigarette would you expect to have the best taste?	Overall	32.4	43.3	12.9	11.4	χ2 (3) = 344.698, p < .001
	Never	25.1	39.7	12.4	22.8	χ2 (6) = 72.980, p < .001
	Ever	32.6	37.8	15.1	14.5	
	Current	35.7	48.1	11.8	4.4	
Which cigarette do you think is most likely to attract your attention?	Overall	22.0	60.9	11.7	5.4	χ2 (3) = 903.219, p < .001
	Never	12.0	68.5	9.8	9.8	χ2 (6) = 47.555, p < .001
	Ever	19.3	62.8	11.6	6.3	
	Current	28.3	56.2	12.6	2.9	
Which cigarette do you think is most dangerous to your health?	Overall	3.5	12.8	61.5	22.2	χ2 (3) = 947.631, p < .001
	Never	5.1	15.2	46.6	33.2	χ2 (6) = 39.503, p < .001
	Ever	3.7	13.4	62.4	20.5	
	Current	2.7	11.4	67.9	18.0	
Which cigarette do you think is least dangerous to your health?	Overall	40.2	16.3	9.0	34.6	χ2 (3) = 312.671, p < .001
	Never	29.3	15.0	10.6	45.1	χ2 (6) = 41.656, p < .001
	Ever	36.2	20.9	11.6	31.3	
	Current	47.6	14.1	6.7	31.6	
Which cigarette would you or someone like you be most willing to try?	Overall	35.0	42.7	6.5	15.8	χ2 (3) = 405.723, p < .001
	Never	23.4	31.8	6.6	38.3	χ2 (6) = 178.481, p < .001
	Ever	33.4	40.0	7.7	18.9	
	Current	41.3	49.3	5.8	3.6	

### Correlates of willingness to try

As shown in Table 2, smoking status and gender were the demographic characteristics most consistently associated with willingness to try a given product style (i.e., unfiltered, filtered cork tipped, or filtered white tipped). Relative to never smokers, current smokers were more likely to select white-tipped (OR = 1.98) and cork-tipped (OR = 3.42) cigarettes, while ever smokers more commonly selected the cork-tipped (OR = 1.96) over the other products. Men were more likely to select cork-tipped (OR = 1.46) and less likely to select white-tipped (OR = 0.55) over other products. Product selections on the basis of taste, attractiveness, and health were also associated with willingness to try a given product. Those willing to try the filtered white-tipped cigarette were more likely to have rated the cigarette as best tasting (OR = 11.10), attracting attention (OR = 17.91), and lowest health risk (OR = 1.94). Similar patterns emerged for willingness to try the filtered cork-tipped and unfiltered cigarettes. The same models were run among smokers only, to allow us to include current brand style (Light/Mild vs. Not) as a covariate. Brand style was not a significant independent predictor of product selection as most likely to try.

**Table 2:** Logistic regression models examining correlates of selecting white-tipped, cork tipped, or unfiltered products, respectively, as ‘most willing to try’

	White			Cork			Unfiltered		
	OR	LCL	UCL	OR	LCL	UCL	OR	LCL	UCL
Smoking Status									
Ever smoker, not current	1.49	0.90	2.48	**1.96**	1.25	3.06	1.03	0.48	2.21
Current smoker	**1.98**	1.22	3.21	**3.42**	2.22	5.27	0.89	0.42	1.89
Never smoker	REF			REF			REF		
Sex									
male	**0.55**	0.37	0.81	**1.46**	1.03	2.07	1.21	0.64	2.28
female	REF			REF			REF		
Taste Best									
White Tip	**11.10**	5.46	22.55	**0.44**	.23	.84	**0.29**	0.09	0.99
Cork Tip	0.80	0.39	1.64	**5.83**	3.35	10.17	0.40	0.13	1.19
Unfiltered	1.49	0.67	3.33	0.83	0.43	1.64	**5.48**	1.95	15.41
No difference	REF			REF			REF		
Attract Attention									
White Tip	**17.91**	5.46	58.80	0.48	0.18	1.28	1.16	0.21	6.47
Cork Tip	1.91	0.60	6.02	**6.24**	2.77	14.07	1.12	0.23	5.40
Unfiltered	1.44	0.41	5.02	**2.54**	1.03	6.26	**7.34**	1.50	35.99
No difference	REF			REF			REF		
Least Dangerous									
White Tip	**1.94**	1.23	3.04	0.98	0.65	1.48	0.75	0.35	1.60
Cork Tip	**0.50**	0.27	0.91	**2.94**	1.77	4.89	0.33	1.92	4.07
Unfiltered	0.76	0.36	1.59	1.00	0.55	1.80	**5.59**	2.29	13.66
No difference	REF			REF			REF		

Note: Models controlled for study site, age, education, race/ethnicity, and perceived smoking risks. Bolded values are significant at *p* < .05

## Discussion

The results of this study show that the perceived risks and benefits of smoking can be manipulated by the presence or absence of a filter and by something as seemingly inconsequential as the color of filter tipping paper. Importantly, these product perceptions were also predictive of a subjects’ willingness to try different product styles. While this finding may seem self-evident, it is important in a number of respects. First, perhaps because of the decades of strategic and deliberate marketing by the tobacco industry that associated lighter packs and product features with low machine delivery levels of tar and nicotine, consumers have come to associate the white tipping paper on cigarette filters with reduced harm from smoking [34–36]. Second, an alarming number of consumers believe that the presence of a cigarette filter offers health protection, judging by their higher rating of perceived danger for unfiltered versus filtered cigarettes, even though convincing evidence from long term epidemiological studies does not support such a claim [12, 14]. Indeed, a 2014 report from the US Surgeon General concluded that the risks of some forms of lung cancer has increased over the past 50 years and the evidence suggests that filters have played a role in this increase [14].

Government agencies charged with regulating tobacco products, such as the U.S. Food and Drug Administration, carry as part of their mission to support public health, including adequate and truthful information about the relative risks and benefits of products. Since the color of the tipping paper used to wrap a filter tip on a cigarette has no meaningful impact on the functionality of a cigarette, but does appear to alter product perceptions, the findings suggest a potential area for regulatory oversight. Australia, for example, recently adopted new product standards that defined the size and style of cigarettes and cigarette packaging in an effort to minimize consumer misperceptions [37].

A similar argument could be made about the use of a filter itself since current scientific evidence does not support its value in improving population health [12, 18]. However, given that virtually all cigarettes sold in the U.S. over the past 40 years have had a filter tip, it would be difficult to simply ban them, as consumers have come to accept the filter as an integral part of the product design. However, at a minimum, manufacturers could be required to inform consumers that the filter on their cigarette offers no meaningful health benefit, and in fact may increase their risk of diseases from compensation and exposure to filter fiber fallout, of which consumers are generally unaware [7, 18].

A limitation of this study is that the recruited samples were not selected to be representative of the U.S. population. However, the findings on consumer perceptions about filters were consistent with other published studies [2, 7], and with internal market research conducted by cigarette manufacturers [3–6, 9], suggesting that they may apply to the larger population. Another limitation of our study was the decision to standardize the brand name used on the test cigarettes (all were Camel), which may have influenced how consumers rated the different product features. However, product selections were similar for those who reported smoking Camel cigarettes as their usual brand (*N* = 91) compared to smokers of other brands (data available on request from corresponding author). Brand name was also masked on the cigarette sticks that participants were shown in this portion of the study, minimizing the potential influence of brand on this particular selection task. This finding suggests that the varied product features (i.e., unfiltered, filtered cork-tipped, and filtered white tipped) were the predominant factor influencing consumer ratings. An important strength of the study is that participants could actually handle the cigarettes to provide more tactile basis for discernment, compared to showing participants product images, as was done in the Borland and Savvas [2] study. The results of the present study are consistent with research conducted with images, as has been found with research on tobacco product warnings [38–41]. As with any relatively new finding, the results of this study need to be replicated and extended to explore how other product features influence consumer product perceptions.

## Conclusion

In summary, study findings demonstrate that consumer product perceptions can be influenced by elements of cigarette design, such as the color of tipping paper used on the filter tip. Government regulators charged with making sure that citizens are not misled about the relative risks and benefits of products need to consider how product design elements influence consumer product perceptions. Design features, such as tipping paper color used on a filter, which have no direct relationship to the health risks or functionality of the product, ought to be standardized so as to avoid misleading consumers.

## References

[1] Wakefield M, Morley C, Horan J, Cummings KM (2002). The cigarette pack as image: new evidence from tobacco industry documents. Tob Control.

[2] Borland R, Savvas S (2014). Effects of stick design features on perceptions of characteristics of cigarettes. Tob Control.

[3] Ferris RP. The influence of brand identification and imagery on subjective evaluation of cigarettes. Rep No RD.1752-C. 1980. Bates 102375094. Retrieved 21 December 2015 from Legacy Tobacco Documents Library, UCSF:. https://industrydocuments.library.ucsf.edu/tobacco/docs/htkc0196.

[4] Johnston ME, Jr. Special Report No. 248. Market Potential of a health Cigarette. Phillip Morris. Bates No. 2040452500–2040452523. ​Retrieved 21 December 2015 from Legacy Tobacco Documents Library, UCSF:. https://industrydocuments.library.ucsf.edu/tobacco/docs/ktjv0125.

[5] Fitzmaurice, R. A. (1979, August 2). Memorandum to H. Cullman and S. Pollack: Marlboro Red 100’s. Philip Morris. Bates No. 2024984161–2024984163. ​Retrieved 21 December 2015 from Legacy Tobacco Documents Library, UCSF:. https://industrydocuments.library.ucsf.edu/tobacco/docs/yzcx0117

[6] Phillip Morris International. Marlboro Lights, approximately 1986. Retrieved 21 December 2015 from Legacy Tobacco Documents Library, UCSF: https://industrydocuments.library.ucsf.edu/tobacco/docs/fswf0189

[7] Hastrup JL, Cummings KM, Swedrock T, Hyland A, Pauly JL (2001). Consumers’ knowledge and beliefs about the safety of cigarette filters. Tob Control.

[8] Czoli CD, Hammond D (2014). Cigarette Packaging: Youth Perceptions of “Natural” Cigarettes, Filter References, and Contraband Tobacco. J Adolesc Health.

[9] Pepples EC. Industry Response to Cigarette/Health Controversy. Retrieved 21 December 2015, from Legacy Tobacco Documents Library, UCSF: https://industrydocuments.library.ucsf.edu/tobacco/docs/jnxd0024

[10] Pauly JL, Mepani AB, Lesses JD, Cummings KM, Streck RJ (2002). Cigarettes with defective filters marketed for forty years: what Philip Morris never told smokers. Tob Control.

[11] Harris B (2011). The intractable cigarette ‘filter problem’. Tob Control.

[12] Thun MJ, Carter BD, Feskanich D, Freedman ND, Prentice R, Lopez AD (2013). 50-year trends in smoking-related mortality in the United States. N Engl J Med.

[13] US Department of Health and Human Services (2010). How Tobacco Smoke Causes Disease: The Biology and Behavioral Basis for Smoking-Attributable Disease: A Report of the Surgeon General.

[14] US Department of Health and Human Services (2014). The Health Consequences of Smoking: 50 Years of Progress: a Report of the Surgeon General.

[15] Brooks DR, Austin JH, Heelan RT, Ginsberg MS, Shin V, Olson SH (2005). Influence of type of cigarette on peripheral versus central lung cancer. Cancer Epidemiol Biomarkers Prev.

[16] Burns DM, Anderson CM, Gray N (2011). Has the lung cancer risk from smoking increased over the last fifty years?. Cancer Causes Control.

[17] Burns DM, Anderson CM, Gray N (2011). Do changes in cigarette design influence the rise in adenocarcinoma of the lung?. Cancer Causes Control.

[18] Kozlowski LT, Dreschel NA, Stellman SD, Wilkenfeld J, Weiss EB, Goldberg ME (2005). An extremely compensatible cigarette by design: documentary evidence on industry awareness and reactions to the Barclay filter design cheating the tar testing system. Tob Control.

[19] Kozlowski LT, O'Connor RJ (2002). Cigarette filter ventilation is a defective design because of misleading taste, bigger puffs, and blocked vents. Tob Control.

[20] Rapp, K. E. (1979, June 4) Memorandum to R.A. Pages: Model III WSC activities in the Salmonella/microsome assay: WSC activity as a function of cigarette filter dilution. - Philip Morris. Bates No. 2501507859–2501507866. ​Retrieved 21 December 2015 from Legacy Tobacco Documents Library, UCSF:. https://industrydocuments.library.ucsf.edu/tobacco/docs/hrgh0115.

[21] Pages, R. A. (1978, May 4) Research report: 6906 annual report-biological effects of smoke. - Philip Morris. Bates No. 2001243600–2001243673. ​Retrieved 21 December 2015 from Legacy Tobacco Documents Library, UCSF:. https://industrydocuments.library.ucsf.edu/tobacco/docs/smky0116.

[22] Levins, R. J. (1984, August 15) Research report: 6908 contribution of the blend components of low tar reference cigarette to CCSC, PAH III yield and Salmonella activity. - Philip Morris. Bates No. 2001291173–2001291198. ​Retrieved 21 December 2015 from Legacy Tobacco Documents Library, UCSF:. https://industrydocuments.library.ucsf.edu/tobacco/docs/lxpd0123.

[23] Novotny TE, Lum K, Smith E, Wang V, Barnes R (2009). Cigarette butts and the case for an environmental policy on hazardous cigarette waste. Int J Environ Res Public Health.

[24] Novotny TE, Hardin SN, Hovda LR, Novotny DJ, McLean MK, Khan S (2011). Tobacco and cigarette butt consumption in humans and animals. Tob Control.

[25] King B, Borland R (2005). What was "light" and "mild" is now "smooth" and "fine": new labelling of Australian cigarettes. Tob Control.

[26] Barnes RL (2011). Regulating the disposal of cigarette butts as toxic hazardous waste. Tob Control.

[27] Schneider JE, Peterson NA, Kiss N, Ebeid O, Doyle AS (2011). Tobacco litter costs and public policy: a framework and methodology for considering the use of fees to offset abatement costs. Tob Control.

[28] Kozlowski LT, Goldberg ME, Yost BA, White EL, Sweeney CT, Pillitteri JL (1998). Smokers' misperceptions of light and ultra-light cigarettes may keep them smoking. Am J Prev Med.

[29] Shiffman S, Pillitteri JL, Burton SL, Rohay JM, Gitchell JG (2001). Smokers' beliefs about "Light" and "Ultra Light" cigarettes. Tob Control.

[30] Cummings KM, Hyland A, Bansal MA, Giovino GA (2004). What do Marlboro Lights smokers know about low-tar cigarettes?. Nicotine Tob Res.

[31] Bansal-Travers M, Hammond D, Smith P, Cummings P (2011). The impact of cigarette pack design, descriptors, and warning labels on risk perception in the U.S. Am J Prev Med.

[32] Hammond D, Doxey J, Daniel S, Bansal-Travers M (2011). Impact of female-oriented cigarette packaging in the United States. Nicotine Tob Res.

[33] Hammond D, Parkinson C (2009). The impact of cigarette package design on perceptions of risk. J Public Health (Oxf).

[34] Yong HH, Borland R, Cummings KM, Hammond D, O'Connor RJ, Hastings G (2011). Impact of the removal of misleading terms on cigarette pack on smokers' beliefs about 'light/mild' cigarettes: cross-country comparisons. Addiction.

[35] Borland R, Fong GT, Yong HH, Cummings KM, Hammond D, King B (2008). What happened to smokers' beliefs about light cigarettes when "light/mild" brand descriptors were banned in the UK? Findings from the International Tobacco Control (ITC) Four Country Survey. Tob Control.

[36] National Cancer Institute (2001). Risks associated with smoking cigarettes having low machine-measured yields of tar and nicotine. Smoking and Tobacco Control Monograph 13.

[37] Hammond D, Reid JL, Driezen P, Boudreau C (2013). Pictorial health warnings on cigarette packs in the United States: an experimental evaluation of the proposed FDA warnings. Nicotine Tob Res.

[38] Department of Health, Australian Government. Plain packaging of tobacco products. https://www.health.gov.au/internet/main/publishing.nsf/Content/tobacco-plain. . Accessed 21 December 2015.

[39] Hammond D, Thrasher JF, Reid JL, Driezen P, Boudreau C, Arillo-Santillán E (2012). Perceived effectiveness of pictorial health warnings among Mexican youth and adults: a population-level intervention with potential to reduce tobacco-related inequities. Cancer Causes and Control.

[40] Thrasher JF, Arillo-Santillán E, Villalobos V, Pérez-Hernández R, Hammond D, Carter J (2012). Can pictorial warning labels on cigarette packages address smoking-related health disparities? Field experiments in Mexico to assess pictorial warning label content. Cancer Causes and Control.

[41] Thrasher JF, Carpenter M, Andrews JO, Gray KM, Alberg AJ, Navarro A (2012). Cigarette warning label policy alternatives and smoking-related health disparities. Am J Prev Med.

